# Construction Notes

**Published:** 1903-12-05

**Authors:** 


### CONSTRUCTION NOTES.

The new Monmouth Hospital, plans of which we recently published, has been opened by Lord Llangattock, who himself contributed £2,000 towards the total cost of £8,000.

Additions have been made to the Hull Victoria Children's Hospital consisting of an enlargement of the out-patients' department, new consulting and dressing rooms, and post-mortem room and mortuary. The mortuary has been provided with stained-glass windows by Mr. W. D. Theaker. The alterations cost about £1,000.

A new ward for children in the Northern Infirmary, Inverness, has been opened, and will memorialise the late Miss Julia Mackintosh, who bequeathed £1,000 for the assistance of sick children. The accommodation is for five cots.

The old Edinburgh Infirmary, more recently used until now as a fever hospital, is to be deserted. The buildings are substantial and the site valuable, and it has not yet been decided what is to be its future fate. The old infirmary has had an interesting and varied career. In 1743 it was used as a military hospital.

